# Co-Delivery of Imiquimod and Curcumin by Nanoemugel for Improved Topical Delivery and Reduced Psoriasis-Like Skin Lesions

**DOI:** 10.3390/biom10070968

**Published:** 2020-06-27

**Authors:** Mohammed S. Algahtani, Mohammad Zaki Ahmad, Ihab Hamed Nourein, Javed Ahmad

**Affiliations:** 1 Department of Pharmaceutics, College of Pharmacy, Najran University, Najran 11001, Saudi Arabia; msalqahtane@nu.edu.sa (M.S.A.); zaki.manipal@gmail.com (M.Z.A.); 2 Department of Clinical Laboratory (Histopathology and Cytology), College of Applied Medical Sciences, Najran University, Najran 11001, Saudi Arabia; ihab213@gmail.com

**Keywords:** nanoemulsion, combinatorial, nanoemulgel, skin permeability, psoriasis-like symptom

## Abstract

The current investigation aimed to improve the topical efficacy of imiquimod in combination with curcumin using the nanoemulsion-based delivery system through a combinatorial approach. Co-delivery of curcumin acts as an adjuvant therapeutic and to minimize the adverse skin reactions that are frequently associated with the topical therapy of imiquimod for the treatment of cutaneous infections and basal cell carcinomas. The low-energy emulsification method was used for the nano-encapsulation of imiquimod and curcumin in the nanodroplet oil phase, which was stabilized using Tween 20 in an aqueous dispersion system. The weak base property of imiquimod helped to increase its solubility in oleic acid compared with ethyl oleate, which indicates that fatty acids should be preferred as the oil phase for the design of imiquimod-loaded topical nanoemulsion compared with fatty acid esters. The phase diagram method was used to optimize the percentage composition of the nanoemulsion formulation. The mean droplet size of the optimized nanoemulsion was 76.93 nm, with a polydispersity index (PdI) value of 0.121 and zeta potential value of −20.5 mV. The optimized imiquimod-loaded nanoemulsion was uniformly dispersed in carbopol 934 hydrogel to develop into a nanoemulgel delivery system. The imiquimod nanoemulgel exhibited significant improvement (*p* < 0.05) in skin permeability and deposition profile after topical application. The in vivo effectiveness of the combination of imiquimod and curcumin nanoemulgel was compared to the imiquimod nanoemulgel and imiquimod gel formulation through topical application for ten days in BALB/c mice. The combination of curcumin with imiquimod in the nanoemulgel system prevented the appearance of psoriasis-like symptoms compared with the imiquimod nanoemulgel and imiquimod gel formulation entirely. Further, the imiquimod nanoemulgel as a mono-preparation slowed and reduced the psoriasis-like skin reaction when compared with the conventional imiquimod gel, and that was contributed to by the control release property of the nano-encapsulation approach.

## 1. Introduction

Imiquimod (IMQ) is a topical immunomodulator that aids in the treatment of superficial basal cell carcinoma, genital warts, and actinic keratosis [1,2]. Indeed, its topical delivery is challenging for formulation scientists due to the poor solubility of IMQ in most pharmaceutical solvents and low skin penetration properties [2]. Despite the efficacy of IMQ, skin reaction and irritation is the commonly reported adverse effect by 75% of patients with topically applied IMQ, resulting in interruptions during treatment [3]. Similarly, patients treated with IMQ can also have mild to severe skin reactions, including pruritus, swelling, vesicles, erosions, erythema, exudation, and crusting [4,5].

Curcumin (CUR) is a polyphenolic compound of natural origin obtained from the rhizome of Curcuma longa. It is widely reported for various therapeutic activities such as anti-inflammatory and anti-cancer activity [6]. CUR modulates the cancer signaling pathway in different malignancies, including melanoma cancer [7]. Besides, CUR increases the efficacy and reduces the adverse effects of various anti-cancer therapeutics when used as an adjuvant/adjunct drug for the combinatorial delivery approach [7,8]. 

Currently, nanotechnology-mediated drug delivery is a leading approach to achieve controlled drug release, co-delivery of primary and adjuvant therapeutics, protecting/minimizing the drug-associated adverse events, and improving the overall drug biopharmaceutical performance [9,10]. For the topical route of administration, the nanocarrier-mediated delivery has a role in reducing the skin irritation associated with the immediate release of some active pharmaceutical ingredients (APIs) by controlling the rate of release [11,12] and enhancing the drug’s skin permeation [13] which results in a more effective treatment outcome and less adverse effects. 

Nanoemulsion (NE) as a drug delivery vehicle is a widely explored method to enhance the biopharmaceutical performance of poorly soluble therapeutics for topical application. It is easy to process and manufacture, with competitive stability thus generating further interest in the development of NE-based topical formulations. NE consists of colloidal oil droplets, ranging in size between 20 and 200 nm, dispersed in an immiscible aqueous medium [14]. Due to the low viscosity of NE, the direct topical application is inconvenient for the patients; therefore, the incorporation of NE into a hydrogel system is favorable. 

The encapsulation of IMQ within an NE system will allow a deeper skin permeation and protects the skin from the direct contact of IMQ. Moreover, the incorporation of IMQ-loaded NE into hydrogel systems (as a secondary vehicle) will result in the precise control of IMQ released from the NE system and minimizes the IMQ-associated skin reactions [11]. 

**Figure 1:**
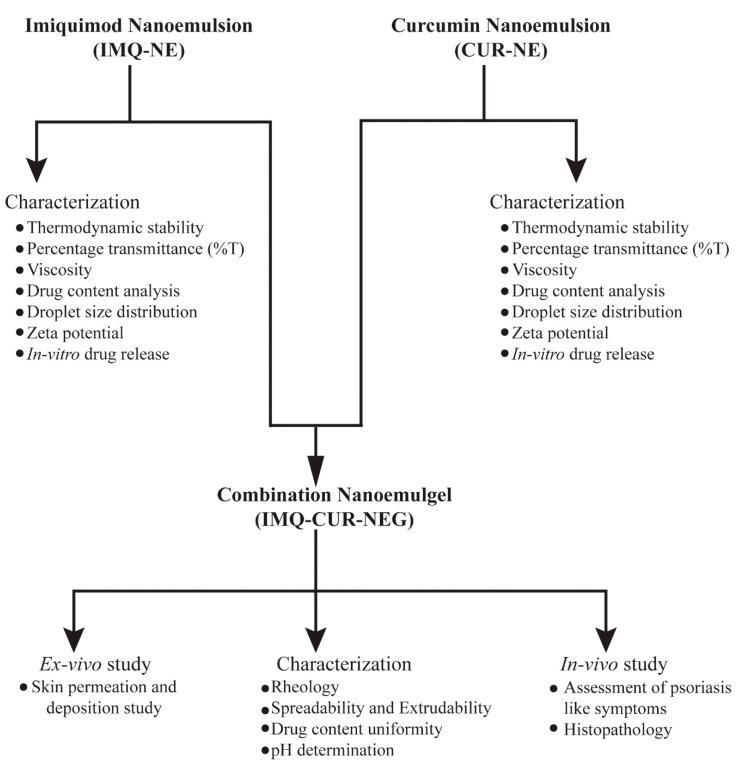
Schematic presentation of experimental methodology illustrating the development of the combination nanoemulgel containing imiquimod and curcumin.

This work aims to improve the topical delivery of IMQ and to reduce the associated adverse effect. The IMQ-NE formulation was optimized to improve the topical delivery of IMQ. Further, CUR-NE was added to the formulation in order to reduce the IMQ-associated skin reaction side effect. Both formulations were incorporated in a hydrogel system to formulate a nanoemulgel (NEG) (schematic presentation of the experimental methodology illustrated in Figure 1). This hydrogel system will hydrate the skin and enhance the performance of the NE-mediated delivery of encapsulated therapeutics.

## 2. Materials and Methods 

### 2.1. Materials 

IMQ (>98.0%) was purchased from Tokyo Chemical Industry, Japan. Oleic acid was purchased from Loba Chem (Mumbai, India). Curcumin (>97.0%), cremophor EL, IPA (isopropyl alcohol), and ethanol were purchased from Sigma Aldrich (Germany). Transcutol HP (diethylene glycol monoethyl ether) was purchased from Gattefose (France). Captex^®^355 and capmul^®^ MCM were provided by Abitec Corporation (USA). Tween 80 (polyoxyethylene sorbitan monooleate), Tween 20 (polyoxyethylene sorbitan monolaurate), PG (propylene glycol), and PEG (polyethylene glycol) 400 were purchased from Merck (Schuchardh, Hokenbrunn, Germany). Water was obtained from a Milli-Q-water purification system (Millipore, Billerica, MA, USA). All the other chemicals were of analytical grade. 

### 2.2. Formulation Design, Optimization and Characterization of Imiquimod Nanoemulsion

#### 2.2.1. Solubility and Phase Behavior Study

In specific oils, surfactants, and co-surfactants, the solubility of IMQ was determined by the addition of an excess of the drug to specified components (2 mL) in stopper vials separately and then mixing with a vortex mixer. The samples were equilibrated in an isothermal water shaker (25 ± 0.5 °C) for 48 h [15]. The equilibrated samples were centrifuged (3000 rpm for 15 min) and the supernatant filtered using a membrane filter (0.45 µm membrane). The filtered samples were analyzed using a UV–visible spectrophotometer at 226 nm.

The phase behavior study was carried out by the construction of phase diagrams [16]. For the construction of phase diagrams, oil and a specific Smix ratio (1:1, 2:1, and 1:2) were uniformly mixed in different ratios (such as 1:9, 2:8, and 3:7) to precisely outline the boundaries of the different phases formed in the phase behavior study by addition of an aqueous phase. The clear, easily flowable, and transparent formulation is visually observed during the phase behavior study and was designated as the region of the NE phase in the phase diagram.

#### 2.2.2. Thermodynamic Stability 

The NE formulations selected from the phase diagram study were centrifuged at 3000 rpm for 30 min. Those formulations that did not show any phase separation were taken for a heating and cooling cycle. Samples were subjected to three cycles between the temperatures of 4 and 45 °C for 48 h to induce stress conditions. Then, the stable formulations were subjected to freeze–thaw cycles. Three freeze–thaw cycles between -21 and +25 °C were carried out to induce stress conditions [17]. The thermodynamically stable NE formulations were selected for further characterization. The composition of the formulations is shown in Table 1.

#### 2.2.3. Percentage Transmittance (%T)

The sample was diluted with distilled water (1:100) and mixed for one minute before analysis. The %T was determined using a UV-spectrophotometer at a wavelength of 638.2 nm [14]. 

#### 2.2.4. Viscosity

The viscosity of the selected IMQ-NE was determined using a Bohlin rotational viscometer (Bohlin Visco 88, Malvern Instruments Ltd., UK). The viscosity of the selected samples was calculated without dilution at room temperature.

#### 2.2.5. Drug Content Analysis

IMQ-NE formulations were diluted in methanol (100 μL of sample dilutes 1000-fold). The IMQ content was quantified using a UV–visible spectrophotometer at 226 nm. 

#### 2.2.6. Analysis of Droplet Size Distribution and Zeta Potential

The droplet size and the zeta potential of IMQ-NE were determined by the dynamic light scattering (DLS) technique using a Zetasizer (Nano ZS90, Malvern Instruments, UK). The samples were diluted with distilled water (1:100) and mixed for one minute before analysis. 

### 2.3. Preparation and Characterization of Curcumin Nanoemulsion 

An accurately weighed quantity of CUR was completely dissolved with the help of vortex mixing in a homogeneous phase of oil and Smix. After that, the aqueous phase was added and subjected to vortex mixing for a few minutes to obtain a clear, transparent, stable, isotropic system in the form of NE similar to the preparation of IMQ-NE described in Section 2.2. The curcumin nanoemulsion (CUR-NE) was characterized for thermodynamic stability, %T, viscosity, drug content analysis, droplet size distribution, and zeta potential, similar to the characterization of IMQ-NE described in Section 2.2. 

### 2.4. In Vitro Drug Release Study

The in vitro release study of IMQ from the selected IMQ-NE formulation and IMQ aqueous suspension were carried out (n = 3) using a dialysis tube (Spectra® dialysis tubing, USA) with a molecular weight cut-off of 12-14 kD [18]. The dialysis tube was kept in diffusion media (500 mL of phosphate buffer saline pH 7.4) containing 1% *w*/*v* albumin and 5% methyl alcohol as a co-solubilizer to maintain sink conditions overnight to saturate the condition. The condition was controlled under constant stirring at 37 °C. A 5 mL sample was collected at specified time intervals: 0, 0.25, 0.5, 1, 2, 4, 6, 8, 10, 12, and 24 h. Each sample was replaced by a fresh medium to maintain the sink conditions. The concentration of IMQ in each sample was determined using a UV–visible spectrophotometer. Furthermore, the release study of the combination of IMQ-NE and CUR-NE (mixed in a 1:1 ratio) was also performed similarly.

### 2.5. Preparation and Characterization of Combination Nanoemulgel 

IMQ containing NEG (IMQ-NEG) and IMQ, along with CUR as a combination NEG (IMQ-CUR-NEG), were prepared. First, Carbopol^®^ 934 (0.5% *w*/*v*) was dispersed into a small portion of water with uniform homogenization. Then, For IMQ-NEG, IMQ-NE was incorporated into the Carbopol 934 to give a final concentration of 0.5% IMQ in the gel. For IMQ-CUR-NEG, a mixture of IMQ-NE and CUR-NE was added to dispersed Carbopol^®^ 934 in a 1:1 ratio to give a final concentration of 0.5% of IMQ and CUR in the gel. 

The formulations were neutralized by adding 2–3 drops of triethanolamine, and the final pH was adjusted to 5.5. Glycerol was added as a plasticizer in the hydrogel. It was kept overnight to remove entrapped air and allow cross-linking of the polymer to convert it into a gel. 

The IMQ-NEG and IMQ-CUR-NEG were characterized regarding their rheology, spreadability, extrudability, content uniformity, and droplet size distribution as well as zeta potential.

#### 2.5.1. Rheology

The rheological properties of IMQ-NEG were studied using a rotational parallel plate viscometer (Bohlin Visco 88, Malvern Instruments Ltd, UK) at 25 ± 0.5 °C. The shear-stress profile (15-200 to 200-15 Pa in 60 steps with 10 s equilibration time at each point) and the thixotropic behavior of IMQ-NEG were determined [14]. The BohlinR6.51.03 software was used for the calculation.

#### 2.5.2. Spreadability and Extrudability

The spreadability of the developed IMQ-NEG formulation was evaluated according to a reported method with slight modifications [19]. The sample (500 mg of IMQ-NEG) was kept at the centre on an acrylic plate, and another plate was put concentrically above it. The spreading diameter of IMQ-NEG was measured. Further, weight was added gradually to the upper plate with a time interval of 60 s. The spreadability factor (SF) was calculated by dividing the value of the maximum spread area by the total weight added. Similarly, the extrudability of IMQ-NEG was evaluated using a method previously described by Ahmad et al. 2019 [14]. An inflatable tube containing the sample (20 g IMQ-NEG) was clamp at the crimped end to prevent any rollback of the sample. The IMQ-NEG was extruded after the removal of the cap. To evaluate the extrudability characteristics of the prepared IMQ-NEG formulations, we observed the force required to extrude a specified amount of gel in a definite time.

#### 2.5.3. Drug Content Uniformity

To ensure the homogeneity of IMQ in the developed NEG formulation, an accurately weighed quantity of the formulation (0.5 g) was taken from three different locations of IMQ-NEG. Each portion of the sample was extracted with 1% albumin in methanol (10 mL) for 30 min. The extracted sample was centrifuged (3000 rpm for 15 min) and the supernatant filtered for the content analysis of IMQ using a UV–visible spectrophotometer. The analysis in terms of the average content and drug percentage was carried out in triplicate [14].

#### 2.5.4. pH Determination

The pH of IMQ-NEG was determined using a digital pH meter (Ezodo, PP201, Taiwan). For this, IMQ-NEG (2.0 g) was dissolved in 25 mL of double-distilled water. The pH electrode was then dipped into the dissolved NEG solution, and the constant reading was noted in triplicate.

### 2.6. Ex Vivo Skin Permeation and Deposition Study

#### 2.6.1. Preparation of Rat Skin

Abdominal skin in full-thickness was obtained from male albino rats (weight 200–250 g). Skin hair was carefully removed using an electric razor. The subcutaneous tissue was surgically removed, and isopropyl alcohol was used to clean away adhered fat at the dermis side. Then, distilled water was used to clean the skin and visually inspected for integrity and stored in a deep freezer until use [20,21].

#### 2.6.2. Drug Permeation and Deposition in the Skin

Ex vivo skin permeation and deposition of IMQ from IMQ-NEG as well as IMQ and CUR from the combination gel (IMQ-CUR-NEG) were studied using albino rat abdominal skin mounted in a Franz diffusion cell. The diffusion area was 1.13 cm^2^, and the capacity of the receiver cell was 10 mL [22].

The receptor compartment was filled with phosphate buffer (pH 5.5) containing 1% *w*/*v* albumin and 5% methyl alcohol as co-solubilizers. Water circulation in the external jacket of the diffusion cell was used to maintain the temperature at 37±0.5ºC. The excised albino abdomen rat skin was mounted between the donor and the receptor compartment and equilibrated at 37 ± 0.5 °C for a sufficient period with constant stirring using a magnetic stirrer. 

The IMQ-CUR gel was prepared as a control sample where IMQ and CUR were dissolved in PG and dispersed in carbopol gel (0.5% *w*/*v*). IMQ-CUR gel and IMQ-CUR-NEG were used to compare the skin permeation and the deposition of IMQ and CUR from the IMQ-CUR-NEG formulation. 

IMQ-CUR-NEG (2 g), IMQ-CUR gel (2 g), and IMQ-NEG (1 g) were introduced into the donor compartment. In a series of time intervals (0, 1, 2, 3, 4, 6, 8, 10, 12, and 24 h), aliquots of 1 mL from each receptor compartment were withdrawn and replaced with 1 mL of the receptor media [20]. The samples were filtered and analyzed using a UV–visible spectrophotometer. The permeability coefficient (Kp), flux (Jss), and enhancement ratio of drug penetration were calculated using a method described by Alvarado et al. [23]. The intercepts of the steady state of the cumulative amount of drug permeated vs. time plot on the x-axis corresponds to the lag time.

The amount of the drugs deposited on the skin layer was determined using the tape stripping technique [24]. Excess drug on the skin surface was washed with PBS at the end of 24 h. Then, 19 mm Scotch^TM^ tape (3M, USA) was used for the tape stripping study. The first strips were discarded as they may have contained the drug remaining on the skin surface after the washing step. The stratum corneum was completely removed by stripping with tape for 15 times on the exposed skin surface. The stripped tapes and the remaining skin samples were soaked in PBS pH 5.5 containing 1% *w*/*v* albumin and 5% methanol overnight, followed by sonication for 60 min to extract the drugs. The extracted samples were analyzed using a UV–visible spectrophotometer. The IMQ-CUR-NEG and IMQ-NEG skin deposition parameters were compared with the IMQ-CUR gel in order to elucidate the effect of the nanoemulsion on the penetration of IMQ and CUR into the skin.

### 2.7. In Vivo Study

#### 2.7.1. Animals 

The animal protocol to carry out the in vivo study was approved by the local institutional animal ethical committee (Najran University, KSA; Ref. no: 23-8-1-19) and followed their guidelines to perform the studies. Male BALB/c mice (6–8 weeks old, weighing 20–30 g) were used for the in vivo investigation and kept under standard laboratory conditions. The animals were retained in polypropylene cages, with free access to standard laboratory diet and water ad libitum. 

#### 2.7.2. Assessment of Psoriasis-Like Symptoms

Animals were divided into three groups, with four mice in each group (n = 4). Group I was treated with IMQ gel, group II was treated with IMQ-NEG, and group III was treated with IMQ-CUR-NEG. All the study groups (Group I, II, and III) received the therapy for 10 consecutive days. The 0.5% *w*/*w* IMQ-gel, 0.5% *w*/*w* IMQ-NEG, and 0.5% *w*/*w* IMQ-CUR-NEG were applied at the amount of 250 mg on the dorsal region of the shaven skin of the area of 6.0 cm^2^ of BALB/C mice with an application frequency of two times a day.

#### 2.7.3. Histopathology

The study was performed to determine the pathological changes after 10 days of skin application of IMQ gel, IMQ-NEG, and IMQ-CUR-NEG. On day 11, the animals were culled, and skin samples (treated area) were collected and fixed in 10% formalin. Later, the collected skin samples were embedded in paraffin, and a microtome was used to prepare tissue sections. Besides, the tissues were stained with hematoxylin and eosin, and the histopathological changes induced in formulation-treated skin were observed under an inverted microscope (at 10× magnification).

### 2.8. Statistical Analysis

Values are expressed as mean ± SD. The data were analyzed using one-way ANOVA followed by the Tukey post hoc test, with values *p* < 0.05 considered significant.

## 3. Results and Discussion

### 3.1. Preparation and Characterization of IMQ-NE and CUR-NE

#### 3.1.1. Solubility Study of IMQ-NE

**Figure 2:**
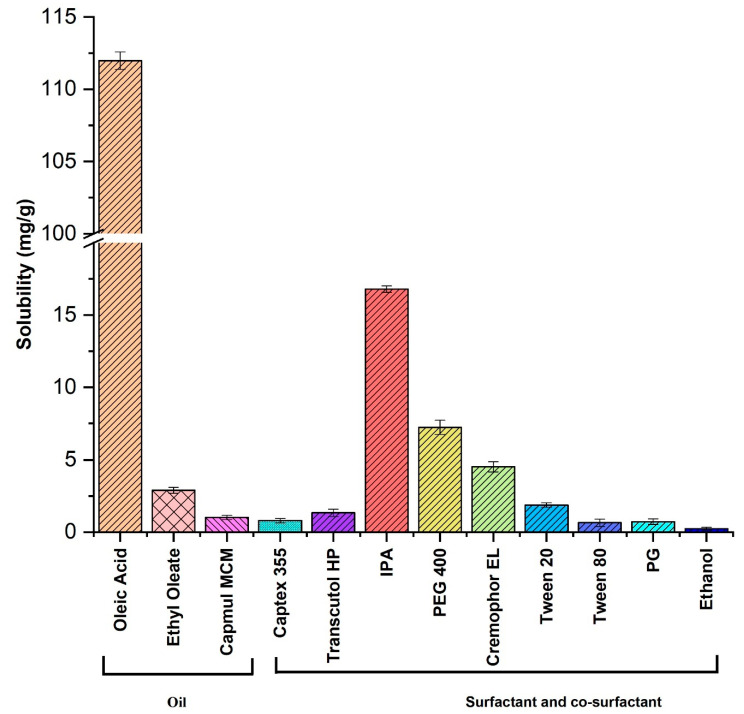
Imiquimod solubility in different oils, surfactants, and co-surfactants (*n* = 3). Solubility of imiquimod in oleic acid is significantly (*p* < 0.05) high compared with other oils, surfactants, and co-surfactants.

The solubility studies of IMQ in different components of NE (oil, surfactant, co-surfactant) were carried out at room temperature. The solubility of IMQ among the tested oil systems was found to be maximum in oleic acid (111.3 ± 0.60 mg/g), followed by ethyl oleate (2.9 ± 0.2 mg/g), capmul^®^ MCM (1.02 ± 0.01 mg/g), and captex^®^355 (0.8 ± 0.01 mg/g) (Figure 2). The weak base property of IMQ helped to increase its solubility in oleic acid oil and convert IMQ into the corresponding salt form [25,26]. Oleic acid is a monounsaturated omega-9 fatty acid with a long chain of 17-carbon atoms and terminal carboxyl group. It is commonly found in various plant and animal products. 

Cremophor EL, Tween 20, Tween 80, PEG 400, PG, Transcutol HP, isopropyl alcohol, and ethanol were tested as surfactant and co-surfactant phases to formulate IMQ-NE. IMQ exhibited moderate solubility in the surfactant and the co-surfactant phases. The best solubility of IMQ in the tested surfactants and co-surfactants was in isopropyl alcohol (16.8 ± 0.23 mg/g), followed by PEG 400 (7.23 ± 0.05 mg/g), Cremophor EL (4.52 ± 0.03 mg/g), and Tween 20 (1.87 ± 0.01 mg/g). It exhibited very low solubility in PG (0.73±0.02 mg/g), Tween 80 (0.65 ± 0.025 mg/g), and ethanol (0.23 ± 0.0 mg/g). 

#### 3.1.2. Optimization of IMQ-NE Exploiting Phase Behavior Study

The emulsification efficiency was assessed for the surfactants and co-surfactants with oleic acid as the oil phase, which provided the maximum drug solubility. Cremophor EL and Tween 20 exhibited significant emulsification efficiency (%T with 51.97 ± 0.44% and 57.29 ± 0.35%, respectively) for the oil phase. However, Cremophor EL in combination with oleic formed a thicker-phase NE compared with Tween 20. Therefore, Cremophor EL was excluded from the study.

**Figure 3:**
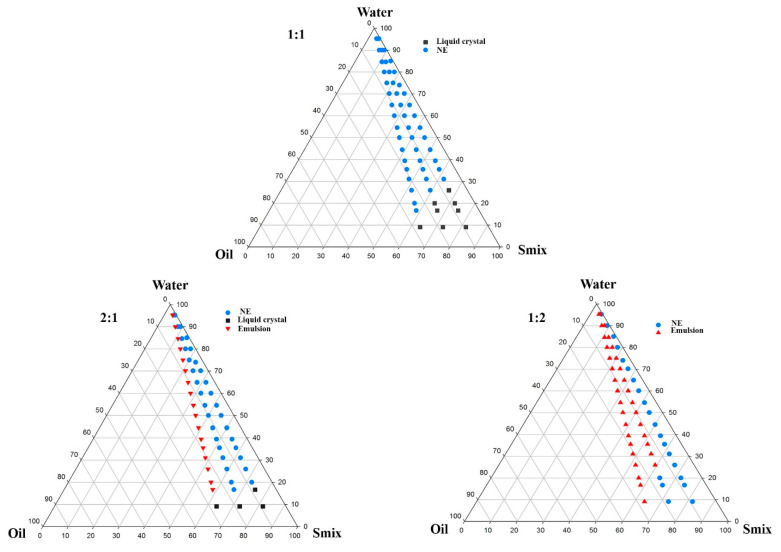
Phase behavior study at Smix ratios of 1:1, 2:1, and 1:2.

Co-surfactants were needed in the formulation development of NE to improve the emulsification efficiency of the surfactant and quickly yield NE by the low-energy emulsification method [23]. Different co-surfactants were mixed with Tween 20 to form the Smix phase. The addition of Transcutol HP to Tween 20 exhibited greater emulsification efficiency (%T with 46.88 ± 0.41) for oleic acid when compared with the other co-surfactants such as ethanol (39.88±0.18), IPA (39.01 ± 0.27), PG (38.96 ± 0.8), and PEG 400 (37.77 ± 0.39). Therefore, Transcutol HP was chosen as the co-surfactant and was added with the surfactant (Tween 20) in different ratios (1:1, 2:1, and 1:2), and aqueous titration was carried out to determine the nano-emulsification region in the phase diagrams (Figure 3). Finally, the concentration of oleic acid, Tween 20, and Transcutol HP were optimized from the phase diagram through a phase behavior study based on the efficiency of the oil and Smix phases to incorporate the maximum quantity of water to form a stable NE (Figure 3).

The specific concentrations of the oil and Smix phases (as shown in Table 1) were uniformly mixed to get a homogenous phase by vortex mixing. Accurate weight of IMQ was dissolved with the help of vortex mixing in the homogenous phase of oil and Smix. After that, the water phase was added, and vortex mixing was carried out for a few minutes to get a clear, transparent, stable isotropic system in the form of an NE with a drug loading concentration of 10 mg/mL.

#### 3.1.3. Characterization of IMQ-NE 

The NE formulations were subjected to thermodynamic stability tests to recognize and prevent metastable formulations. They were subjected to heating–cooling cycles, centrifugation tests, and freeze–thaw cycle stability tests. All the tested formulations passed the thermodynamic stability tests with no physical changes such as creaming, cracking, and coalescence. The percent content of IMQ in all the selected NE (NE1–NE4) was quantified by UV–visible spectrophotometric analysis. The IMQ content was above 99% for all NE formulations (Table 1). 

The spectrophotometric determination of %T showed optically clear NE formulations with oil droplets in a very fine dispersion [27]. The NE compositions with a higher %T were expected to have a droplet size in the nano-dimension. The %T was in the range of 91.07 ± 0.7 for NE3 and 98.88 ± 0.03 for NE4. 

The viscosity measurement of the selected formulations (NE1–NE4) was carried out at ambient temperature (25ºC). The viscosity measurement is presented in Table 1. The formulation NE2 exhibited maximum viscosity (63.82 ± 0.62 cP), whereas NE1 exhibited minimum viscosity (55.26 ± 0.81 cP). The viscosity of IMQ-loaded NE (NE1-NE4) remained constant with an increase in the rate of shear (>15 s^−1^) and exhibited Newtonian fluid behavior [28]. This is indicative of the unrestricted flow of droplets in the direction of the shearing force [29]. 

For the optimum topical drug delivery using an NE formulation, NE globules should have a large surface area to volume ratio with a polydispersity index (PdI) value less than 1 [30,31]. The smallest oil droplet size (76.93 nm) was in NE4, and the largest oil droplet size (197.1 nm) was in NE1. An increase in oil droplet size was correlated with an increase in the oleic acid concentration (10% to 15%) and a decrease in the surfactant concentration (seen with a change in the Smix ratio 2:1 to 1:2), as shown in Table 1. This finding indicates that the Smix ratio has a significant influence on the droplet size of IMQ-NE. It also emphasizes that the surfactant concentration is responsible for the changes in oil droplet size, while the co-surfactant concentration is responsible for the PdI of the NE droplets.

The surface charge magnitude has a direct impact on the stability of NE since electrical repulsive forces between NE droplets minimize the chance of coalescence [32,33]. The droplet surface of the selected formulations (NE1–NE4) showed a negative zeta potential (Table 1). The NE2 formulation showed the highest negative charge (−35.8), while the NE1 formulation showed the lowest negative charge (10.9) (Table 1). The presence of anionic groups in fatty acids and glycols in the NE components led to this negative charge [33]. NE globules with a negative surface charge are capable of deeper skin penetration by keeping the droplet size at its minimum [34,35]. 

**Figure 4:**
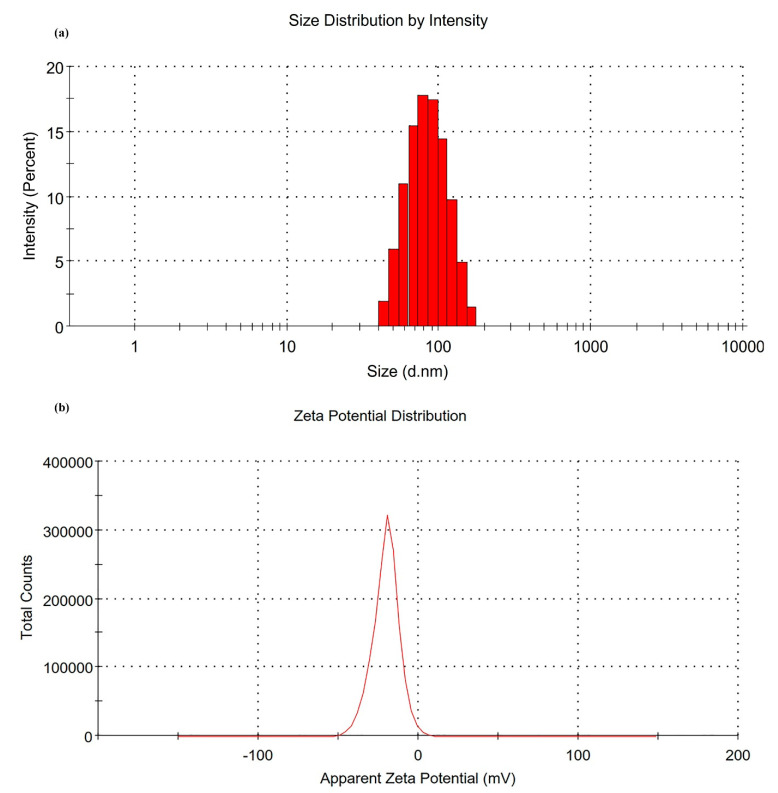
(**a**) Droplet size distribution of NE4. (**b**) Zeta potential distribution of NE4.

The droplet size, PdI, and zeta potential characterizations helped with the selection of the optimum formulation to proceed with the in vitro and in vivo studies. The results of NE4 were found to be very promising for IMQ skin delivery due to the droplet size dimension < 100 nm with PdI < 0.2. The NE4 showed more precision and uniformity in the droplet size distribution (76.93 nm) and had the lowest PdI (0.121) (Figure 4a). NE4 had a highly negative surface charge with a zeta potential value of −20.5 mV (Figure 4b). This suggests that the NE4 formulation has the least chance of flocculation/coalescence or Ostwald ripening [33,34,36]. NE4 was selected for the in vitro release studies as well as for the incorporation of the NE4 formulation into a hydrogel system.

#### 3.1.4. Characterization of CUR-NE

CUR-NE was prepared with a drug loading concentration of 15 mg/mL and characterized for thermodynamic stability (passed all the stress tests for stability), %T (99.12 ± 0.21), viscosity (125.48 ± 1.54 cP), %drug content analysis (99.26 ± 0.13), droplet size distribution (10.57 nm with PdI 0.094), and zeta potential (−18.7 mV), similar to what was carried out for IMQ-NE in the previous investigation of our research lab [37].

### 3.2. In Vitro Drug Release Study

**Figure 5:**
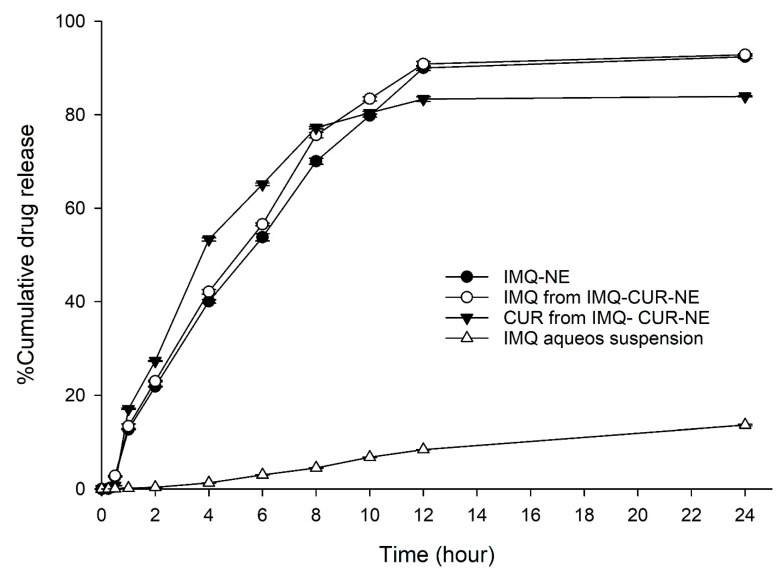
In vitro drug release profile of IMQ from different formulation (IMQ-NE, IMQ-curcumin (CUR)-NE) and CUR from IMQ-CUR-NE in comparison to aqueous suspension of IMQ.

The in vitro release of IMQ from the optimized formulation (NE4) was carried out using the dialysis bag technique. The release study (n = 3) was carried out for 24 h. The release of IMQ from the IMQ-NE formulation was compared to the release of IMQ from aqueous suspension. The release of IMQ from IMQ-NE formulation exceeded the IMQ release from the aqueous suspension (Figure 5). After 24 h, only around 11% of the IMQ was released from the aqueous suspension, while 92% of IMQ was released from IMQ-NE (Figure 5). 

As the aim of this work was to formulate a combined formulation for the co-delivery of IMQ and CUR through the NEG system, the release of IMQ and CUR from the combined formulation system (IMQ-NE mixed with CUR-NE in 1:1 ratio) was evaluated. The combination of the two NE formulations (IMQ-NE and CUR-NE) did not affect the release of IMQ (92.84 ± 0.27%) and CUR (83.94 ± 0.13) (Figure 5).

The different kinetic models, such as zero order, first order, Higuchi, and Korsmeyer–Peppas were applied to predict the release kinetics and the diffusion behavior of IMQ from IMQ-NE and IMQ-CUR-NE and CUR from IMQ-CUR-NE (Table 2) [23]. The best-fitting release kinetic model was indicated by the value of the correlation coefficient (r^2^) (Table 2). Overall, the best curve fitting representing the IMQ and CUR release from the respective NE formulation followed the Korsmeyer–Peppas model (highest value of r^2^) with Fickian diffusion (*n* < 0.45), as shown in Table 2. This indicates the minimal possible interaction between the drug molecules and NE components [38].

### 3.3. Preparation and Characterization of IMQ-NEG and IMQ-CUR-NEG

IMQ-NEG was prepared by dispersing IMQ-NE in 0.5% *w*/*v* Carbopol® 934 gel to obtain a 0.5% *w*/*w* concentration of IMQ in the gel. IMQ-CUR-NEG also was prepared by dispersing IMQ-NE and CUR-NE in a 1:1 ratio in 0.5% *w*/*v* Carbopol® 934 gel to obtain a 0.5% *w*/*w* concentration of IMQ and CUR. The effect of IMQ-NE incorporation into the hydrogel system in terms of its mean droplet size and PdI was evaluated. The results showed no significant change in the mean droplet size (78.39 nm) or PdI (0.254) after the incorporation of IMQ-NE into the gel system.

**Figure 6:**
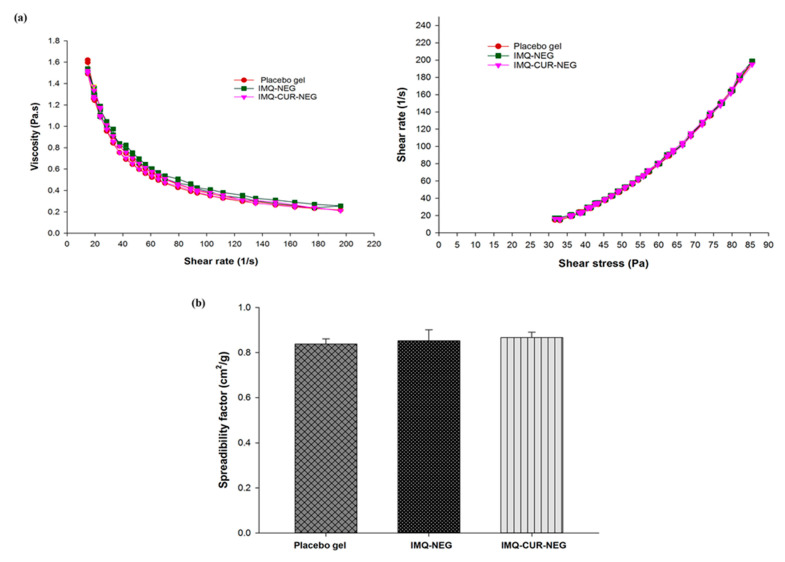
(**a**) Rheological behavior of placebo gel, IMQ-NEG, and IMQ-CUR-NEG. (**b**) The spreadability factors of the placebo gel, IMQ-NEG, and IMQ-CUR-NEG are nearly equal and the difference in the value of the spreadability factor between these three gels was found to be statistically insignificant (*p* > 0.05) (*n* = 3).

Figure 6a shows the rheological profile (viscosity versus shear rate; shear rate versus shear stress) of the placebo gel (without the incorporation of IMQ-NE and CUR-NE), IMQ-NEG, and IMQ-CUR-NEG. All samples exhibited similar rheological behavior. The rheological behavior of the placebo gel, IMQ-NEG, and IMQ-CUR-NEG underwent gel-to-sol transformation and exhibited shear-thinning on the application of shear stress (Figure 6a). However, gel recovery was slow upon the removal of stress, suggesting non-Newtonian and pseudoplastic behavior (shear-thinning) with thixotropic properties [39]. Such rheological behaviors of NEG are convenient for topical application [40]. 

The spreading factor is an important quality control test for the topical application of semisolid preparations [41]. The spreading factors of the placebo gel (0.82 ± 0.02 cm^2^/g), IMQ-NEG (0.85 ± 0.04 cm^2^/g), and IMQ-CUR-NEG (0.87 ± 0.02 cm^2^/g) were found to be equivalent and did not differ (Figure 6b). Further, all formulations exhibited good extrudability from the container tube for patient-friendly applications. The drug content uniformity of IMQ and CUR in the NEG formulations was ≈99% (Table 3). 

The optimum pH range for the topical application of a semisolid formulation should be in the range of 5–6 to minimize skin irritation [42]. The pH of IMQ-NEG and IMQ-CUR-NEG was in the range of 5.5, which is close to the pH of the acid mantle of human skin, which should not induce skin irritation.

### 3.4. Ex Vivo Skin Permeation and Deposition Study

The ex vivo drug permeation and deposition studies were performed on excised albino rat skin mounted in a Franz diffusion cell. The skin deposition of IMQ from IMQ-NEG was 1205.2 ± 21.4 µg/cm^2^ and from IMQ-CUR-NEG was 1367.6 ± 13.2 µg/cm^2^, which is about five-fold higher than the skin deposition of IMQ deposited from the IMQ-CUR gel (243.01 ± 5 µg/cm^2^) (Table 3). CUR skin deposition from IMQ-CUR-NEG was 5178.4 ± 22 µg/cm^2^, about nine-fold higher than CUR deposition from the IMQ-CUR gel (570.9 ± 1 µg/cm^2^) (Table 3). The cumulative amount of drug permeated vs. time plot is illustrated in Supplementary Figure S1.

The percutaneous drug flux (Jss) of the IMQ from IMQ-NEG (0.042 ± 0.01) and IMQ-CUR-NEG (0.071 ± 0.01) was about ten-fold the Jss of IMQ from the IMQ-CUR gel (0.004 ± 0.001) (Table 3). The Jss of the CUR from IMQ-CUR-NEG (36.47±0.01) was four-fold higher than the Jss of CUR from the IMQ-CUR gel (8.365 ± 0.03). 

The permeability coefficient (Kp) of the IMQ from IMQ-NEG (0.0047 x 10^-3^) and IMQ-CUR-NEG (0.0806 x 10^-3^) was significantly higher than the Kp of IMQ from the IMQ-CUR gel (0.45 x 10^-6^) (Table 3). The Kp of CUR from IMQ-CUR-NEG (3.04 x 10^-3^) was significantly higher than the Kp of CUR from the IMQ-CUR gel (0.7 x 10^-3^). Therefore, the permeation enhancement ratios (ER) of IMQ obtained from IMQ-NEG and IMQ-CUR-NEG were 9.54 and 16.19, respectively. Further, the enhancement ratio of CUR obtained from IMQ-CUR-NEG was found to be 4.36. 

### 3.5. In Vivo Study

**Figure 7:**
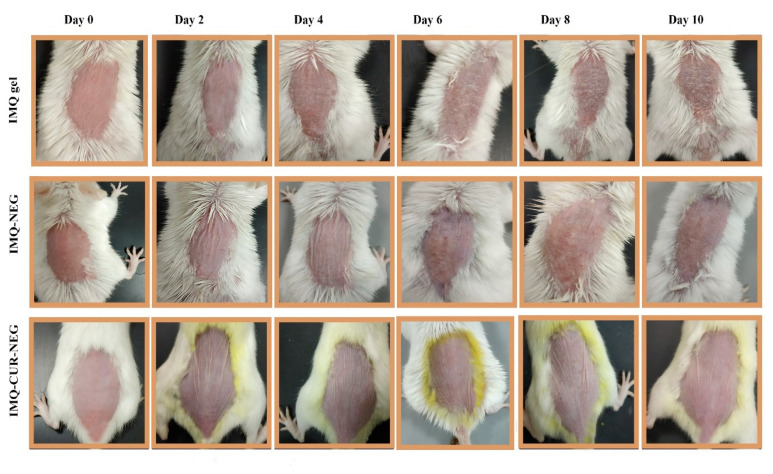
Assessment of psoriasis-like symptoms after the topical application of IMQ gel, IMQ-NEG, and IMQ-CUR-NEG for ten days.

IMQ is a widely used drug for the treatment of different types of skin cancer and known to induce psoriasis-like symptoms in BALB/c mice [1,3]. The aim here was to enhance the transdermal delivery of IMQ using NEG, as well as to reduce the reported skin irritation induced by IMQ through the co-delivery of IMQ with CUR in NEG. The effectiveness of the IMQ-CUR-NEG formulation was compared to the IMQ gel and IMQ-NEG formulation after topical application. Figure 7 shows the development of psoriasis-like symptoms during the experimental period.

Psoriasis-like symptoms like scaling, redness, and skin thickening started to appear on BALB/c mice treated with IMQ gel from the second day and progressively worsened until the tenth day of the experiment (Figure 7). BALB/c mice treated with IMQ-NEG exhibited the delayed appearance of psoriasis-like symptoms (Figure 7). These delayed symptoms are correlated with the controlled release of IMQ from the IMQ-NEG formulation. BALB/c mice treated with IMQ-CUR-NEG did not exhibit any significant appearance of psoriasis-like symptoms like scaling, redness, and skin thickening (Figure 7). The disappearance of psoriasis-like symptoms was correlated with the anti-psoriatic activity of CUR [43]. 

**Figure 8:**
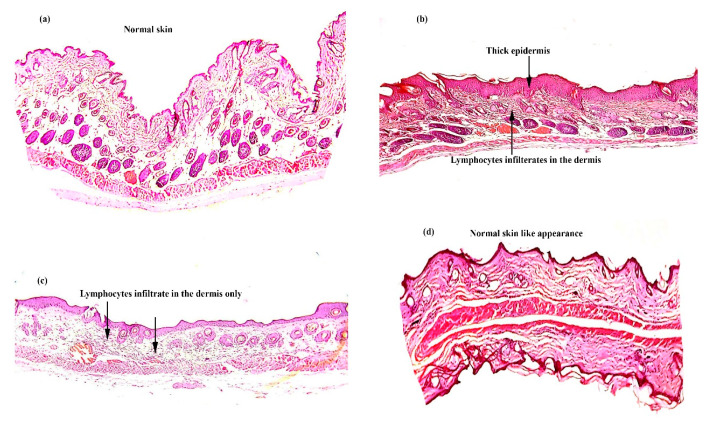
Histopathology of the skin of normal BALB/c mice after ten days of topical treatment: (**a**) untreated skin; (**b**) skin treated with the IMQ gel; (**c**) skin treated with IMQ-NEG; and (**d**) skin treated with IMQ-CUR-NEG (at 10x magnification).

The BALB/c mice were sacrificed on day 11. The treated skin area was collected for histopathology. Figure 8 shows samples of skin that had been treated with the IMQ gel, IMQ-NEG, and IMQ-CUR-NEG as well as the untreated skin sample. The untreated skin shows a regular epidermis and dermis. The skin treated with the IMQ gel shows hyperkeratosis, parakeratosis, acanthosis, and epidermal infiltrates. The skin treated by IMQ-NEG showed similar signs but less thickening of the epidermis layer compared with the skin treated with the IMQ gel. Further, a few irregularities were noticed in the epidermis layer of the skin treated with IMQ-NEG. The skin treated with IMQ-CUR-NEG showed similar characteristics to the untreated normal skin, and insignificant infiltrates were observed (Figure 8).

The histopathology results are in parallel with the in vivo results. The application of the IMQ gel induced aggressive psoriasis-like symptoms (Figure 7 and Figure 8), which is related to the immediate release of IMQ, leading to the commonly associated skin irritation [3,5]. The controlled release of IMQ from IMQ-NEG reduced and delayed this skin irritation (Figure 7 and Figure 8). The co-delivery of IMQ with CUR in IMQ-CUR-NEG resulted in healthy skin with no irritation, similar to the untreated skin. 

IMQ is amongst the most commonly used chemotherapeutic agents for skin cancer [44,45]. Furthermore, interconnected pathways observed in cancer pathophysiology minimize the feasibility of monotherapy. Indeed, the use of combination therapy has been demonstrated to be effective in preliminary clinical trials. CUR in combination is suggested to improve the therapeutic effectiveness of chemotherapeutics by inhibiting the ABC efflux transports in conjunction with other medications. Besides, it was also advocated that CUR in combination with another therapeutics arrests the progression of the cell cycle, induces apoptosis, inhibits the expression of anti-apoptotic proteins, inhibits the multiple cell survival signaling pathways and their cross-communication, and influences the modulation of immune responses [6,44,46]. In addition, it has been widely investigated and is emerging as a topical therapeutic for different types of skin cancer [45,46]. All of these properties make CUR a promising chemo-sensitizing agent in skin cancer for combination therapy with IMQ [44]. The in vivo results of the IMQ-CUR-NEG combined formulation after topical administration in BALB/c mice showed that CUR helps to modulate the immune responses induced by IMQ and neutralizes its psoriasis-like effects on skin after topical application.

## 4. Conclusions

The use of oleic acid (fatty acid) successfully increases the nanoencapsulation of the weakly basic imiquimod to formulate a nanoemulsion system for controlled topical delivery through the skin. The combinatorial approach with CUR was found to be effective in preventing the adverse skin reactions that are frequently associated with the topical use of imiquimod. This result likely occurs due to the encapsulation of imiquimod into the nanoemulgel delivery system, which resulted in controlled drug release when in contact with the skin as well as the co-delivery of curcumin leading to the complete suppression of psoriasis-like symptoms after the topical application.

## Figures and Tables

**Table 1:** Composition and characterization of different imiquimod nanoemulsions (IMQ-NE).

Formu-Lation	%Oil	%Smix		%Water	%T	η (cp)	%Drug Content	Mean Droplet Size (nm)	PDI	ζ (mv)
		Tween 20 (%)	Trans-Cutol HP (%)							
NE_1_	15.0	18.33	36.67	30.0	93.18 ± 0.217	55.26 ± 0.81	99.73 ± 0.09	197.1	0.249	−10.9
NE_2_	10.0	20.0	40.0	30.0	98.51 ± 0.180	63.82 ± 0.62	99.30 ± 0.22	147.7	0.245	−35.8
NE_3_	15.0	36.67	18.33	30.0	91.07 ± 0.698	60.25 ± 0.64	99.83 ± 0.07	122.5	0.242	−26.2
NE_4_	10.0	40.0	20.0	30.0	98.88 ± 0.036	62.98 ± 0.65	99.24 ± 0.13	76.93	0.121	−20.5

**Table 2:** In vitro drug release kinetics of IMQ and CUR from NE formulations.

Figure	Korsmeyer-Peppas Model		Zero-Order	First-Order	Higuchi Model
	r^2^	n	r^2^	r^2^	r^2^
IMQ-NE	0.9986	0.35	0.9863	0.9855	0.9707
IMQ from IMQ-CUR-NE	0.9963	0.344	0.9792	0.9800	0.9724
CUR from IMQ-CUR-NE	0.9934	0.290	0.9258	0.9843	0.9846
IMQ aqueous suspension	0.9258	0.48	0.7896	0.9807	0.8844

**Table 3:** Characterization of different gel formulations (IMQ-NEG, IMQ-CUR-NEG, and IMQ-CUR gel).

Parameters	IMQ-NEG	IMQ-CUR-NEG	IMQ-CUR Gel
Spreadability factor (cm^2^/g)	0.85 ± 0.04	0.87 ± 0.02	-
Drug content uniformity(mg)	99.8 ± 0.1	99.48 ± 0.01	-
		98.85 ± 0.01 (CUR)	-
pH	5.54 ± 0.03	5.51 ± 0.02	-
Drug deposited in skin (µg/cm^2^)	1205.190 ± 21.40	1367.646 ± 13.21	243.01 ± 5.90
		5178.442 ± 22.23 (CUR)	570.86 ± 12.01 (CUR)
Cumulative amount of drug permeated (µg)	1.14 ± 0.01	1.34 ± 0.02	0.123 ± 0.01
		989.333 ± 3.97 (CUR)	226.853 ± 0.98 (CUR)
J_ss_ (µg/cm^2^ .h) *	0.042 ± 0.01	0.071 ± 0.01	0.004 ± 0.001
		36.47 ± 0.01 (CUR)	8.365 ± 0.03 (CUR)
Lag time (h)	0.72 ± 0.016	0.934 ± 0.01	2.92 ± 0.02
		0.668 ± 0.01 (CUR)	1.70 ± 0.06 (CUR)
Permeability coefficient (K_p_ x 10^-3^) **	0.0047	0.0806	0.45
		3.04 (CUR)	0.7 (CUR)
ER ***	9.54 ± 0.79	16.19 ± 1.36	1
		4.36 ± 0.03 (CUR)	1

* Jss = transdermal flux, calculated from the cumulative amount of drug permeated vs. time. ** Permeability coefficient was calculated as Kp = Jss /C_0_ (C_0_ = the initial drug concentration in the donor compartment). *** ER = enhancement ratio, ratio of transdermal flux from nanoemulgel to gel.

## Supplementary Materials

The following are available online at https://www.mdpi.com/2218-273X/10/7/968/s1, Figure S1: (a) Cumulative amount of IMQ permeated from IMQ-NEG, IMQ-CUR-NEG and IME-CUR gel (*n* = 3). (b) Cumulative amount of CUR permeated from IMQ-CUR-NEG and IME-CUR gel (*n* = 3).
