# Supplementary material for: Co-Delivery of Imiquimod and Curcumin by Nanoemugel for Improved Topical Delivery and Reduced Psoriasis-Like Skin Lesions
Source: Biomolecules. 2020 Jun 27;10(7):968. doi: 10.3390/biom10070968 (PMC7407235; doi:10.3390/biom10070968)
Supplement: Supplementary file 1 [file biomolecules-10-00968-s001.pdf]

Supplementary Materials

Co-delivery of Imiquimod and Curcumin by Nanoemugel for Improved Topical Delivery and Reduced Psoriasis-like Skin Lesions

Mohammed S. Algahtani [1], Mohammad Zaki Ahmad [1], Ihab Hamed Nourein [2] and Javed Ahmad [1,*]

[1] Department of Pharmaceutics, College of Pharmacy, Najran University, Najran 11001, Saudi Arabia; msalqahtane@nu.edu.sa (M.S.A.); zaki.manipal@gmail.com (M.Z.A.); ihab213@gmail.com (I.H.N.)

[2] Department of Clinical Laboratory (Histopathology and Cytology), College of Applied Medical Sciences, Najran University, Najran 11001, Saudi Arabia

* Correspondence: jaahmed@nu.edu.sa or jahmad18@gmail.com; Tel.: +966-17542-8744

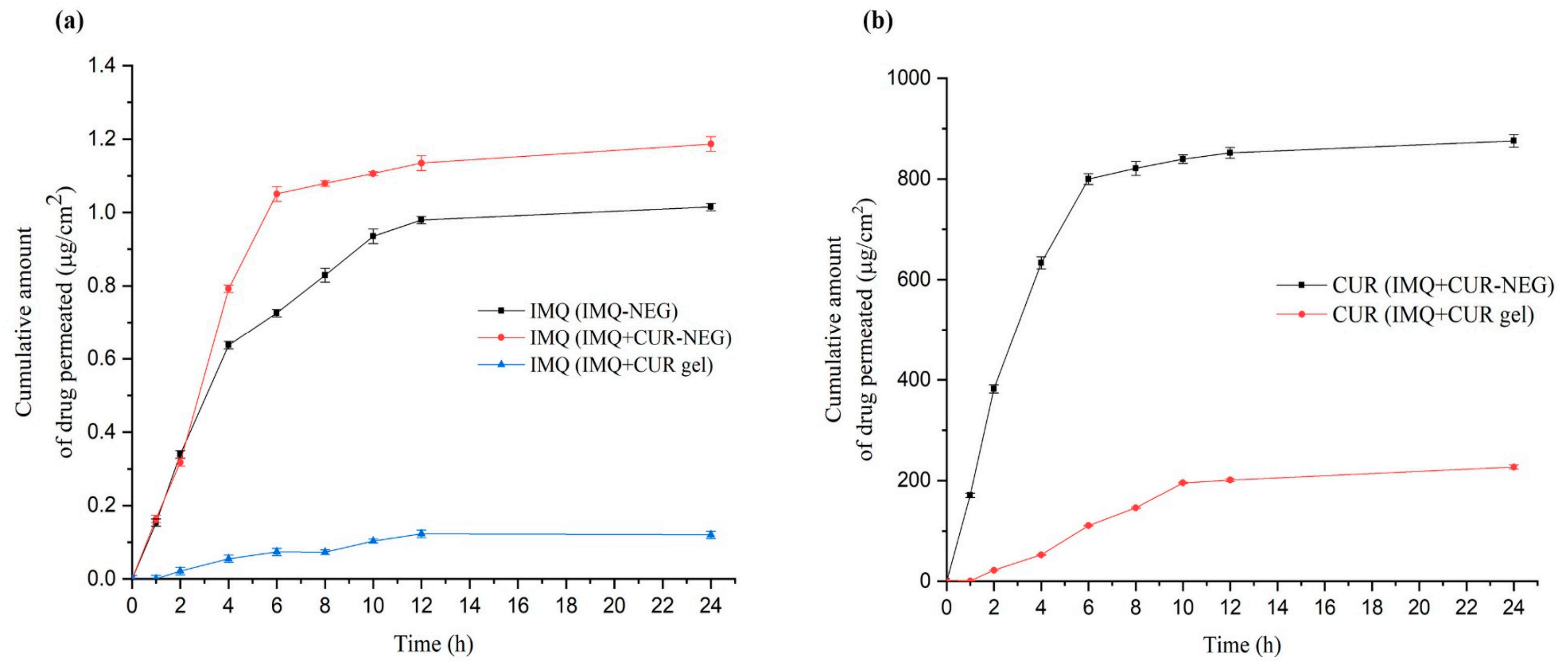

Figure S1. (a) Cumulative amount of IMQ permeated from IMQ-NEG, IMQ-CUR-NEG, and IME-CUR gel (n=3). **(b)** Cumulative amount of CUR permeated from IMQ-CUR-NEG and IME-CUR gel ($n = 3$).
